# Supplementary material for: Modeling glioblastoma heterogeneity as a dynamic network of cell states
Source: Mol Syst Biol. 2021 Sep 16;17(9):e10105. doi: 10.15252/msb.202010105 (PMC8444284; doi:10.15252/msb.202010105)
Supplement: Supplementary file 5 — Source Data for Figure 3 [file MSB-17-e10105-s001.zip › Figure3A_sourcedata/GSEA_3065/hallmarks_state4.GseaPreranked.1623416296068/HALLMARK_ADIPOGENESIS.html]

Details for gene set HALLMARK\_ADIPOGENESIS[GSEA]

|  || Dataset | state4 |
| Phenotype | NoPhenotypeAvailable |
| Upregulated in class | na\_pos |
| GeneSet | HALLMARK\_ADIPOGENESIS |
| Enrichment Score (ES) | 0.27707928 |
| Normalized Enrichment Score (NES) | 1.2087978 |
| Nominal p-value | 0.099585064 |
| FDR q-value | 0.21408774 |
| FWER p-Value | 0.88 |
Table: GSEA Results Summary

  

Fig 1: Enrichment plot: HALLMARK\_ADIPOGENESIS      
 Profile of the Running ES Score & Positions of GeneSet Members on the Rank Ordered List

  

| PROBE | GENE SYMBOL | GENE\_TITLE | RANK IN GENE LIST | RANK METRIC SCORE | RUNNING ES | CORE ENRICHMENT || 1 | SPARCL1 |  |  | 17 | 0.416 | 0.0371 | Yes |
| 2 | SCP2 |  |  | 83 | 0.261 | 0.0516 | Yes |
| 3 | UQCR11 |  |  | 107 | 0.245 | 0.0713 | Yes |
| 4 | UQCRQ |  |  | 158 | 0.218 | 0.0841 | Yes |
| 5 | GPX4 |  |  | 183 | 0.210 | 0.1003 | Yes |
| 6 | ECH1 |  |  | 245 | 0.187 | 0.1083 | Yes |
| 7 | ATP5PO |  |  | 272 | 0.180 | 0.1213 | Yes |
| 8 | ETFB |  |  | 315 | 0.172 | 0.1310 | Yes |
| 9 | DECR1 |  |  | 340 | 0.167 | 0.1431 | Yes |
| 10 | CMBL |  |  | 345 | 0.166 | 0.1583 | Yes |
| 11 | CHCHD10 |  |  | 363 | 0.162 | 0.1711 | Yes |
| 12 | SOD1 |  |  | 396 | 0.152 | 0.1805 | Yes |
| 13 | SORBS1 |  |  | 406 | 0.151 | 0.1935 | Yes |
| 14 | VEGFB |  |  | 414 | 0.149 | 0.2067 | Yes |
| 15 | NDUFB7 |  |  | 445 | 0.144 | 0.2157 | Yes |
| 16 | RNF11 |  |  | 498 | 0.137 | 0.2203 | Yes |
| 17 | NDUFA5 |  |  | 516 | 0.134 | 0.2304 | Yes |
| 18 | APOE |  |  | 569 | 0.129 | 0.2343 | Yes |
| 19 | PDCD4 |  |  | 796 | 0.104 | 0.2075 | Yes |
| 20 | DDT |  |  | 820 | 0.102 | 0.2136 | Yes |
| 21 | TALDO1 |  |  | 830 | 0.101 | 0.2218 | Yes |
| 22 | PHYH |  |  | 865 | 0.098 | 0.2257 | Yes |
| 23 | COX7B |  |  | 872 | 0.098 | 0.2341 | Yes |
| 24 | SDHC |  |  | 880 | 0.097 | 0.2423 | Yes |
| 25 | GHITM |  |  | 938 | 0.093 | 0.2419 | Yes |
| 26 | LAMA4 |  |  | 985 | 0.089 | 0.2430 | Yes |
| 27 | SSPN |  |  | 1013 | 0.087 | 0.2470 | Yes |
| 28 | YWHAG |  |  | 1016 | 0.087 | 0.2550 | Yes |
| 29 | NMT1 |  |  | 1049 | 0.085 | 0.2579 | Yes |
| 30 | ACO2 |  |  | 1077 | 0.083 | 0.2615 | Yes |
| 31 | ITSN1 |  |  | 1093 | 0.082 | 0.2669 | Yes |
| 32 | MDH2 |  |  | 1114 | 0.081 | 0.2715 | Yes |
| 33 | UQCR10 |  |  | 1128 | 0.080 | 0.2771 | Yes |
| 34 | CAT |  |  | 1179 | 0.077 | 0.2764 | No |
| 35 | SLC25A1 |  |  | 1230 | 0.075 | 0.2754 | No |
| 36 | RAB34 |  |  | 1330 | 0.069 | 0.2659 | No |
| 37 | ACLY |  |  | 1333 | 0.069 | 0.2722 | No |
| 38 | PGM1 |  |  | 1389 | 0.067 | 0.2697 | No |
| 39 | QDPR |  |  | 1398 | 0.066 | 0.2748 | No |
| 40 | TOB1 |  |  | 1439 | 0.065 | 0.2744 | No |
| 41 | ACAA2 |  |  | 1493 | 0.062 | 0.2718 | No |
| 42 | PFKL |  |  | 1526 | 0.061 | 0.2724 | No |
| 43 | COX8A |  |  | 1583 | 0.059 | 0.2689 | No |
| 44 | ABCA1 |  |  | 1654 | 0.057 | 0.2630 | No |
| 45 | IDH1 |  |  | 1691 | 0.055 | 0.2625 | No |
| 46 | UCP2 |  |  | 1737 | 0.054 | 0.2603 | No |
| 47 | NDUFAB1 |  |  | 1773 | 0.053 | 0.2597 | No |
| 48 | DLD |  |  | 1906 | 0.049 | 0.2430 | No |
| 49 | LIFR |  |  | 1972 | 0.048 | 0.2370 | No |
| 50 | CRAT |  |  | 2077 | 0.045 | 0.2243 | No |
| 51 | UBQLN1 |  |  | 2094 | 0.044 | 0.2259 | No |
| 52 | BAZ2A |  |  | 2102 | 0.044 | 0.2290 | No |
| 53 | TST |  |  | 2118 | 0.043 | 0.2307 | No |
| 54 | CPT2 |  |  | 2137 | 0.043 | 0.2319 | No |
| 55 | ACOX1 |  |  | 2140 | 0.043 | 0.2357 | No |
| 56 | ACADM |  |  | 2224 | 0.041 | 0.2261 | No |
| 57 | G3BP2 |  |  | 2318 | 0.039 | 0.2147 | No |
| 58 | MTCH2 |  |  | 2357 | 0.038 | 0.2122 | No |
| 59 | COQ9 |  |  | 2372 | 0.038 | 0.2135 | No |
| 60 | BCL2L13 |  |  | 2466 | 0.036 | 0.2018 | No |
| 61 | ALDH2 |  |  | 2519 | 0.034 | 0.1966 | No |
| 62 | IDH3G |  |  | 2528 | 0.034 | 0.1986 | No |
| 63 | ECHS1 |  |  | 2628 | 0.032 | 0.1856 | No |
| 64 | AGPAT3 |  |  | 2652 | 0.032 | 0.1849 | No |
| 65 | PHLDB1 |  |  | 2761 | 0.029 | 0.1701 | No |
| 66 | DBT |  |  | 2764 | 0.029 | 0.1726 | No |
| 67 | CDKN2C |  |  | 2822 | 0.028 | 0.1660 | No |
| 68 | MCCC1 |  |  | 2825 | 0.028 | 0.1684 | No |
| 69 | TANK |  |  | 2890 | 0.027 | 0.1606 | No |
| 70 | MGST3 |  |  | 2993 | 0.025 | 0.1464 | No |
| 71 | MAP4K3 |  |  | 3032 | 0.024 | 0.1425 | No |
| 72 | LPL |  |  | 3121 | 0.023 | 0.1304 | No |
| 73 | PEMT |  |  | 3269 | 0.019 | 0.1083 | No |
| 74 | PRDX3 |  |  | 3387 | 0.016 | 0.0908 | No |
| 75 | UCK1 |  |  | 3411 | 0.015 | 0.0885 | No |
| 76 | RIOK3 |  |  | 3439 | 0.014 | 0.0855 | No |
| 77 | BCL6 |  |  | 3469 | 0.013 | 0.0820 | No |
| 78 | SUCLG1 |  |  | 3497 | 0.012 | 0.0788 | No |
| 79 | FAH |  |  | 3563 | 0.010 | 0.0692 | No |
| 80 | RETSAT |  |  | 3588 | 0.009 | 0.0662 | No |
| 81 | NDUFS3 |  |  | 3667 | 0.006 | 0.0541 | No |
| 82 | UBC |  |  | 3691 | 0.006 | 0.0509 | No |
| 83 | CMPK1 |  |  | 3693 | 0.005 | 0.0512 | No |
| 84 | COX6A1 |  |  | 3753 | 0.003 | 0.0419 | No |
| 85 | DNAJB9 |  |  | 3929 | -0.006 | 0.0140 | No |
| 86 | SLC27A1 |  |  | 4082 | -0.014 | -0.0094 | No |
| 87 | IDH3A |  |  | 4114 | -0.016 | -0.0129 | No |
| 88 | UQCRC1 |  |  | 4194 | -0.022 | -0.0237 | No |
| 89 | PLIN2 |  |  | 4233 | -0.024 | -0.0275 | No |
| 90 | COL4A1 |  |  | 4362 | -0.033 | -0.0452 | No |
| 91 | MYLK |  |  | 4481 | -0.043 | -0.0603 | No |
| 92 | ABCB8 |  |  | 4591 | -0.051 | -0.0732 | No |
| 93 | PTCD3 |  |  | 4607 | -0.052 | -0.0707 | No |
| 94 | HIBCH |  |  | 4615 | -0.052 | -0.0668 | No |
| 95 | CYC1 |  |  | 4648 | -0.055 | -0.0667 | No |
| 96 | DHRS7 |  |  | 4779 | -0.066 | -0.0815 | No |
| 97 | SLC25A10 |  |  | 4807 | -0.068 | -0.0794 | No |
| 98 | ADCY6 |  |  | 4818 | -0.069 | -0.0744 | No |
| 99 | TKT |  |  | 4829 | -0.070 | -0.0693 | No |
| 100 | ARL4A |  |  | 4864 | -0.073 | -0.0678 | No |
| 101 | PPP1R15B |  |  | 4999 | -0.083 | -0.0817 | No |
| 102 | IMMT |  |  | 5023 | -0.084 | -0.0774 | No |
| 103 | SLC19A1 |  |  | 5083 | -0.090 | -0.0783 | No |
| 104 | JAGN1 |  |  | 5115 | -0.093 | -0.0745 | No |
| 105 | MRPL15 |  |  | 5251 | -0.106 | -0.0863 | No |
| 106 | GADD45A |  |  | 5297 | -0.109 | -0.0831 | No |
| 107 | ATL2 |  |  | 5335 | -0.112 | -0.0784 | No |
| 108 | REEP5 |  |  | 5378 | -0.116 | -0.0741 | No |
| 109 | POR |  |  | 5407 | -0.119 | -0.0672 | No |
| 110 | DHRS7B |  |  | 5412 | -0.120 | -0.0563 | No |
| 111 | ESYT1 |  |  | 5416 | -0.120 | -0.0453 | No |
| 112 | CAVIN1 |  |  | 5591 | -0.143 | -0.0598 | No |
| 113 | ELOVL6 |  |  | 5663 | -0.154 | -0.0566 | No |
| 114 | ITGA7 |  |  | 5715 | -0.163 | -0.0493 | No |
| 115 | RTN3 |  |  | 5793 | -0.181 | -0.0445 | No |
| 116 | CD151 |  |  | 5981 | -0.230 | -0.0528 | No |
| 117 | DHCR7 |  |  | 6083 | -0.287 | -0.0418 | No |
| 118 | APLP2 |  |  | 6087 | -0.288 | -0.0146 | No |
| 119 | ATP1B3 |  |  | 6201 | -0.449 | 0.0101 | No |
Table: GSEA details [plain text format]

  

Fig 2: HALLMARK\_ADIPOGENESIS: Random ES distribution      
 Gene set null distribution of ES for **HALLMARK\_ADIPOGENESIS**
